# New implications for prion diseases therapy and prophylaxis

**DOI:** 10.3389/fnmol.2024.1324702

**Published:** 2024-03-04

**Authors:** Fangzhou Liu, Wenqi Lü, Ling Liu

**Affiliations:** ^1^Department of Neurology, West China Hospital, Sichuan University, Chengdu, Sichuan, China; ^2^Department of Psychiatry and Mental Health Center, West China Hospital, Sichuan University, Chengdu, Sichuan, China

**Keywords:** prion diseases, neurodegenerative disorders, therapeutics, prophylaxis, Creutzfeldt-Jakob disease

## Abstract

Prion diseases are rare, fatal, progressive neurodegenerative disorders that affect both animal and human. Human prion diseases mainly present as Creutzfeldt-Jakob disease (CJD). However, there are no curable therapies, and animal prion diseases may negatively affect the ecosystem and human society. Over the past five decades, scientists are devoting to finding available therapeutic or prophylactic agents for prion diseases. Numerous chemical compounds have been shown to be effective in experimental research on prion diseases, but with the limitations of toxicity, poor efficacy, and low pharmacokinetics. The earliest clinical treatments of CJD were almost carried out with anti-infectious agents that had little amelioration of the course. With the discovery of pathogenic misfolding prion protein (PrPSc) and increasing insights into prion biology, amounts of novel technologies have attempted to eliminate PrPSc. This review presents new perspectives on clinical and experimental prion diseases, including immunotherapy, gene therapy, small-molecule drug, and stem cell therapy. It further explores the prospects and challenge associated with these emerging therapeutic approaches for prion diseases.

## 1 Introduction

Prion diseases are a unique group of rare, fatal, and transmissible neurodegenerative disorders, mainly characterized by progressive dementia, myoclonus, and ataxia (Zerr, 2022b). Prion diseases include scrapie in sheep, bovine spongiform encephalopathy in cattle, chronic wasting disease in cervids, and human prion diseases (Manka et al., 2022). Traditionally, human prion diseases include different clinical conditions that present as Creutzfeldt-Jakob disease (CJD), fatal familial insomnia (FFI), Gerstmann-Sträussler-Scheinker disease (GSS) and kuru. ALL four manifestations also can be sporadic, inherited, infective or iatrogenic, according to etiological categories (Collinge, 2001). CJD is the most common in human prion diseases, the annual incidence of CJD is 1–2 cases per million people worldwide (Mead et al., 2022). Despite being rare, CJD has captured public attention because of the long incubation period in the genetic form (gCJD) and the outbreak of variants (vCJD) caused by eating infected beef.

Among neurodegenerative disorders, prion diseases are notorious for their exceptionally progressive clinical course with an average survival of 5 months (Pocchiari et al., 2004). To date, there is no effective therapy for prion diseases, although much effort has been made in this field. The earliest attempts to treat CJD, performed when the agent was generally assumed to be a virus, were carried out with antiviral drugs, such as aciclovir or amantadine, but both were unsuccessful (Herishanu, 1973; David et al., 1984). *In vivo* or *vitro* experiments show that antifungal amphotericin B (Masullo et al., 1992), antibacterial doxycycline (Forloni et al., 2009; Vetrugno et al., 2014), antiparasitic quinacrine (Barret et al., 2003) and anticoagulant pentasane polysulfate (Bone et al., 2008) can prolong the life cycle of cells or animals, but there is no obvious effect in human experiments, either in case reports or systematic observations. The first randomized clinical study in patients with CJD investigated Flupirtine maleate, a centrally acting and nonopioid analgesic, demonstrated cytoprotective activity in cell culture experiments induced by prion protein fragments (Perovic et al., 1997). But it did not show efficacy in terms of survival time in patients with CJD (Otto et al., 2004). Further randomized trials were demonstrated that neither quinacrine nor doxycycline (Geschwind et al., 2013; Haïk et al., 2014) showed obvious effect in the delay in disease progress.

While an effective treatment is not currently available, prion diseases are well understood at the molecular level, with all evidence pointing to the pathophysiological mechanism of PrPSc (Brandner et al., 1996), a conformational change of the normal host encoded prion protein (PrPC) (Prusiner et al., 1990; Prusiner, 1998). Preventing the conversion of native PrPC into its misshapen form or reducing the deposition of pathological PrPSc in cells represents a uniquely attractive therapeutic target. The winner of the Nobel Prize for Physiology and Medicine Prusiner (1991) proposed that the underlying replication mechanism of prions is unprecedented, and it is perhaps not surprising that anti-infectious agents (viral, bacterial, fungal, and parasitic) remain minimally effective during the course of prion diseases modification. In recent years, scientists have focused on eliminating PrP as the target of prion diseases therapies, including immunotherapy, gene therapy, small-molecular drug, and stem cell therapy. This article aims to review the current paradigms of clinical and experimental research on prion diseases, discussed promising directions for future research and the problems of translating laboratory findings into clinically useful therapies.

## 2 Immunotherapies against prion diseases

**Figure 1:**
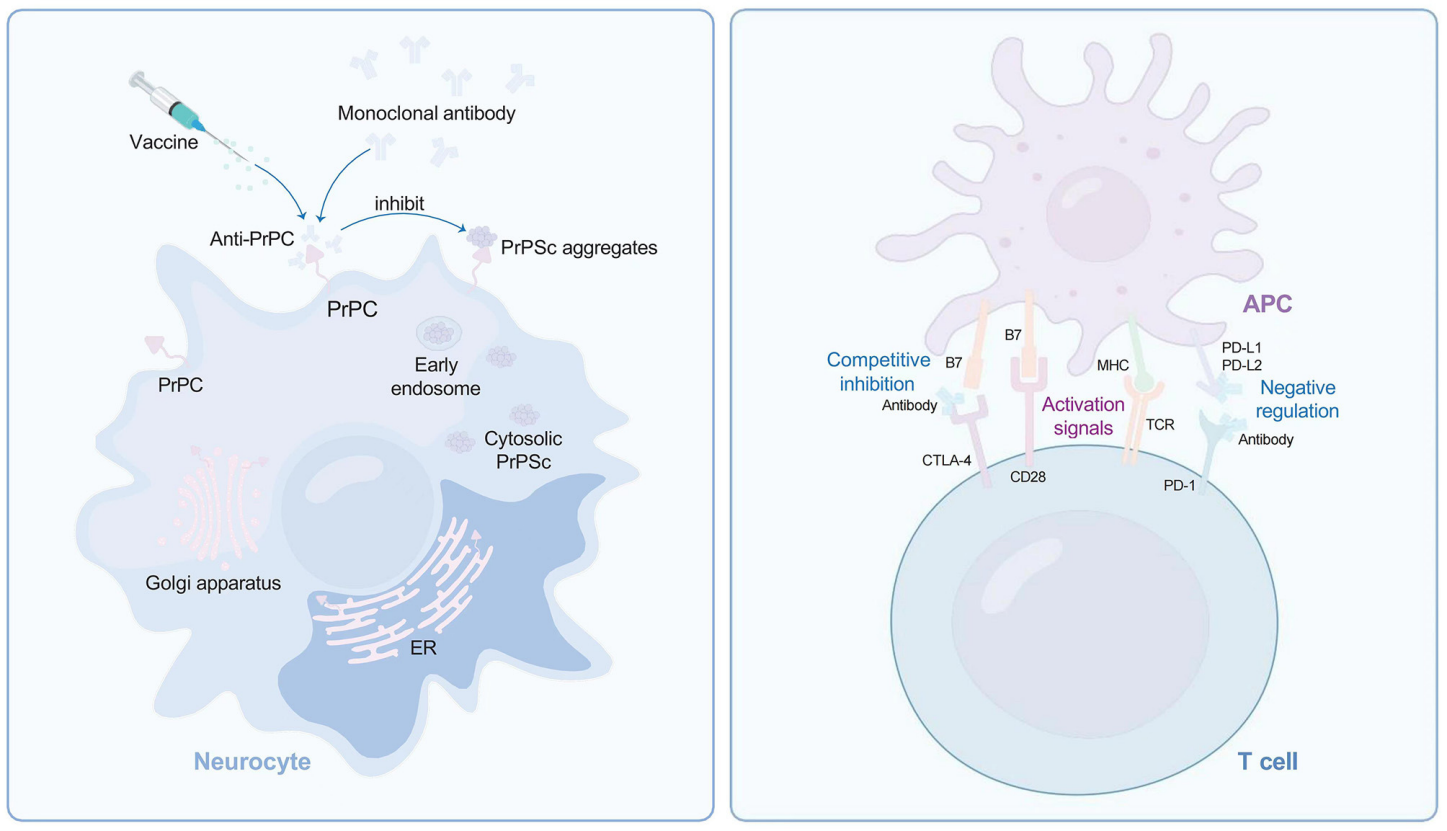
Immunotherapies against prion diseases. The conversion of PrPC to PrPSc probably begins on the plasma membrane and enters the cytoplasm through endocytosis. Vaccine and anti-PrP monoclonal antibody preventing PrPC conversion into harmful PrPSc **(left)**. T-cells can be activated only when TCR and CD28 are activated simultaneously **(right)**. To prevent excessive activation of T-cells, T-cells themselves express CTLA-4, competing with CD28 to block T-cell activation. Prions utilizes the negative regulation characteristics of the human immune system to promote CTLA-4 expression, thereby tolerant to body's immunity. PD-1, like CTLA-4, can attenuate T cell activation. Antibodies direct to CTLA-4 or PD1 can activate T cells to overcome immune escape mechanisms in prion diseases. PrPC, cellular prion protein; PrPSc, pathogenic misfolding prion protein; TCR, T-cell receptor; MHC, major histocompatibility complex; APC, antigen presenting cell; PD1, programmed cell death 1; CTLA4, cytotoxic T lymphocyte-associated antigen 4.

In 2001, Dr. Prusiner hypothesized that “most neurodegenerative diseases are related to the accumulation of abnormal protein within the affected brain regions” (Prusiner, 2001). Therefore, some pharmacological chaperones (vaccines, monoclonal antibodies, and etc.) can be designed to stabilize the folded PrPC, thus preventing its conversion into the disease-associated isoform (Figure 1, left) (Nicoll et al., 2010). With the ongoing progress of research, recent studies have revealed that the progressive activation of neuroinflammation cells in the brain play a crucial role in neurodegenerative diseases. Consequently, utilizing immune checkpoint blockade, including PD-1 antibodies, PD-L1 antibodies, CTLA-4 antibodies, and others, emerges as a strategy to combat prion diseases by enhancing the adaptive immune system (Figure 1, right). The following will provide a detailed introduction to these immune methods.

### 2.1 Active immunization against prion diseases

The active immune activation in prion diseases poses is hampered by self-tolerance. While the three-dimensional conformations of PrPSc and PrPC differ, their amino acid sequences are identical; hence, the pathological accumulation of PrPSc does not induce a classical immune response. Over the years, numerous research groups have investigated strategies to overcome self-tolerance. The first method uses modified PrPs, including truncated, dimers, heterologous, and crosslinked PrP peptides. Immunogens such as PrP131–150, PrP211–230, PrP98–127 or PrP158–187 peptides elicited robust immune responses in mice, and effectively delaying scrapie (Souan et al., 2001; Bachy et al., 2010). Study have shown that immunizing prion-infected mice with recombinant bovine PrP induces the production of anti-PrP autoantibodies and prolongs the incubation times in mice (Ishibashi et al., 2007). The second approach involves DNA vaccine that encodes specific PrP sequences to enhance immune response (Alexandrenne et al., 2010; Han et al., 2011). However, it remains undetermined whether these DNA vaccines could effectively prevent prion infection. The third strategy is employing bacterial or viral vectors to bypass immune tolerance. Sigurdsson et al. (2002) overcame this obstacle by vaccinating mice with recombinant PrPSc mixed with a powerful adjuvant, heat-killed Mycobacterium. The forth method, though mucosal vaccination, overcomes immune tolerance. Utilizing an attenuated Salmonella vaccine has been shown to protect white-tailed deer from chronic wasting disease (Goñi et al., 2015; Taschuk et al., 2017). The fifth method involves the development of vaccines specifically targeting PrPSc. The identification of the YYR motif, YML motif in β-sheet 1, and the rigid loop linking β-sheet 2 to α-helix 2 revealed regions exposed in the misfolded conformation of the protein (Marciniuk et al., 2014; Taschuk et al., 2014). These vaccines targeting PrPSc indeed induced sustained PrPSc-specific antibody responses, but whether this vaccination approach provides protection against prion disease remains unknown (Ma and Ma, 2020).

These active immunotherapies are highly effective in animal models of neurodegenerative diseases, with no obvious side effects in these animals. However, the first clinical trial of active immunization in patients with Alzheimer's disease (AD) was related to unacceptable toxicity (meningoencephalitis) (Hock et al., 2002; Dodart et al., 2003). Given these immune based therapies have great potential efficacy for such devastating diseases, this approach should not be prematurely abandoned, and further investigation should be warranted to maximize efficacy and minimize serious adverse events before they can be safely applied to human. These immune-based treatment approaches may be applicable to high-risk populations (e.g., carriers of prion protein gene (*PRNP*) mutations and medical personnel with potential exposure risk) in the future.

### 2.2 Prion protein monoclonal antibodies have demonstrated promising results in clinical research programs

Compared with other neurodegenerative disorders, prion diseases can be zoonoses, which breaks species barriers and transmits between different mammalian species, and exist in multiple strain types as a cloud or ensemble of sub-species (Collinge and Clarke, 2007). Therefore, previous research targeted the reduction of pathogenic PrPSc which risks the development of drug resistance due to strain selection. Ghaemmaghami et al. (2009) showed that mice treated with quinacrine initially had decreased PrPSc levels; however, this reduction was transient and PrPsc levels recovered rapidly, and a similar phenomenon was observed in cultured differentiated prion-infected neuroblastoma cells. They proposed that quinacrine eliminates a specific subset of PrPSc conformers, but results in the survival of drug-resistant prion conformations, which may explain the low efficacy of quinacrine or other anti-prion drugs. Conversely, agents that bind to PrPC may prove effective against all prion strains (Collinge, 2016). Additionally, initiating treatment prior to the loss of major neurons and irreversible secondary neurodegeneration holds promise as a secondary prophylaxis among *PRNP* mutation carriers, as well as individuals exposed to prions through medical, surgical, or laboratory events.

Both cell culture and animal experiments have supported the use of anti-PrP monoclonal antibodies to inhibit the incorporation of PrPC into propagating prions and delay the progression of prion disease (Enari et al., 2001; Peretz et al., 2001; White et al., 2003; Song et al., 2008). Mead et al. (2022) reported the first in-body treatment of six CJD patients intravenous with a humanized monoclonal antibody to cellular prion protein (PRN100), and the control group was based on the historical data of matched with untreated patients. This study reported that PRN100 can access the brain without clinically significant adverse effects. Brain autopsy report of two patients showed no evidence of neurotoxicity and suggested that PRN100 may help clear disease-related PrPSc; however, all patients showed progressive neurological decline on serial assessments with the Medical Research Council Prion Disease Rating scale (Mead et al., 2022). Similarly, anti- Aβ antibodies (lecanemab and aducanumab), which have received the Food and Drug Administration (FDA) approval or accelerated approval for patients with AD, effectively reduce Aβ deposition on positron emission tomography scans but show inefficacy in addressing cognitive and neurological decline (Terao and Kodama, 2024). These results are very encouraging and long-awaited; however, given the limited number of patients included and the use of historical controls, we cannot determine whether PRN100 has changed the course of the disease; these results may be considered preliminary results (Zerr, 2022a).

### 2.3 Immune modulators: a lesson from cancer immunotherapy

Targeted immune modulator therapy via gene or drug ablation has become a popular topic in recent years. Immune checkpoints, such as programmed cell death 1 (PD-1), cytotoxic T lymphocyte-associated antigen 4 (CTLA-4), and lymphocyte activation gene 3 (LAG-3), are important mechanisms for preventing the immune system overactivation (Zang, 2018), the overexpression of these immune checkpoints establish an immunosuppressive state called tolerance toward body cells, which is the root cause of the immune system's inability to clear tumor cells. Immune checkpoint inhibitors have been successfully employed to treat specific cancer types. Examples include PD-1 antibodies (Pembrolizumab, Nivolumab, Cemiplimab), PD-L1 antibodies (Atezolizumab, Avelumab, Durvalumab), CTLA-4 antibodies (Ipilimumab), and LAG-3 antibodies (Relatlimab). James P. Allison and Tasuku Honjo were awarded the 2018 Nobel Prize for the development of a revolution in cancer medicine (The Nobel Prize in Physiology or Medicine, 2018).

In analogy to cancer, prion diseases have the problem of immune tolerance, which may be the reason why prion diseases are difficult to cure. Therefore, it is assumed that immune checkpoint blockade could be considered a potential means to overcome immune escape mechanisms and efficiently remove pathological protein aggregates in neurodegenerative diseases. Baruch et al. (2016) showed that PD-1 blockade treatment reduced the cerebral Aβ plaque load, and improved cognitive performance in two mouse models of AD. Furthermore, emerging evidence indicates that the absence of LAG3 delays the α-synuclein-induced loss of dopamine neurons in a mouse model of Parkinson's disease (PD) (Mao et al., 2016). However, in prion diseases, targeted PD-1 blockade and LAG-3 blockade have no significant influence on prion deposition and the course of the disease (Liu et al., 2018; Obst et al., 2018). Currently, preclinical evidence for this approach in neurodegenerative diseases remains contradictory. With the growing application of immune checkpoint blockade in numerous common cancers, it is conceivable that some cancer patients might develop neurodegenerative diseases with advancing age. Analyzing the disease progression in these patients, including a neurological assessment of autopsied brains, may offer crucial insights into these pivotal questions (Liu and Aguzzi, 2019).

## 3 Targeted gene therapies

### 3.1 Antisense oligonucleotides, a powerful story of a scientist with *PRNP* mutation

**Figure 2:**
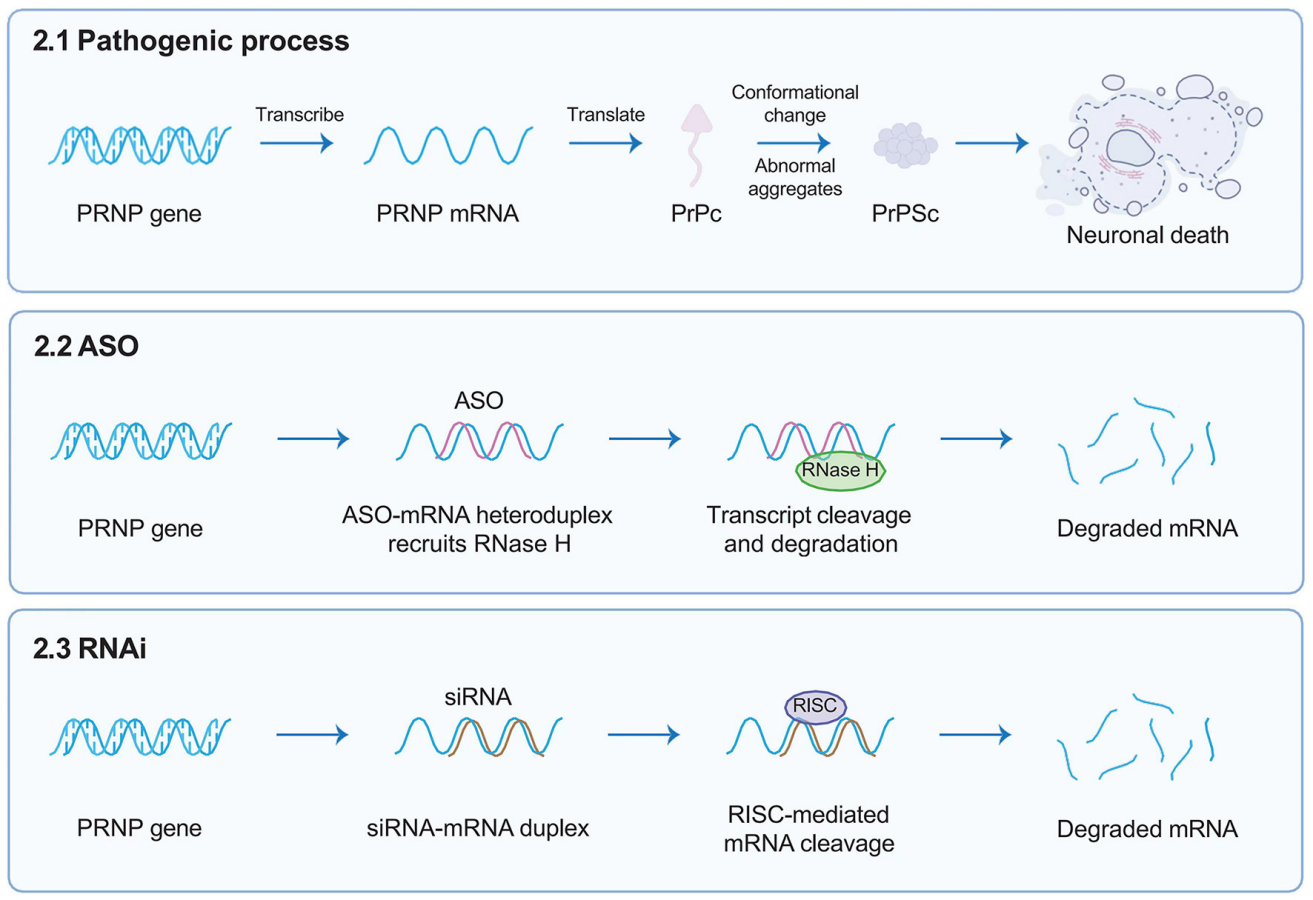
Targeted gene therapies. This figure briefly describes target gene therapies such as ASOs and RNAi, designed to target the human *PRNP* RNA sequence. ASOs are short, single-stranded, synthetic oligonucleotides designed to bind the target mRNA by Watson–Crick base pairing and degrade RNA-DNA complexes through RNase H; whereas siRNAs interact with RISC that can mediate the mRNA degradation of targeted gene transcription. Both reducing the overall amount of PrPC in the brain. ASOs, antisense oligonucleotides; RNAi, RNA interference; *PRNP*, prion protein gene; RISC, RNA-induced silencing complex; PrPC, cellular prion protein.

Antisense oligonucleotides (ASOs) are short, single-stranded, synthetic oligonucleotides designed to bind the target mRNA by Watson–Crick base pairing and degrade RNA-DNA complexes through RNase H, consequently reducing the levels of encoded deleterious proteins (Figure 2) (Rinaldi and Wood, 2018). ASOs-mediated therapies, such as nusinersen for the treatment of spinal muscular atrophy (SMA) and eteplirsen for Duchenne muscular dystrophy, have received approval from the United States FDA. These therapies have the potential to significantly impact the treatment of other genetic neurological conditions. Genetic prion diseases (gCJD, GSS, and FFI) are attributed to mutations in the *PRNP* gene, constituting ~15% of all human prion diseases. Currently, there are no specific treatments for genetic prion diseases, the diagnosis of genetic incurable diseases is devastating for individuals and families.

Vallabh, a carrier of *PRNP* mutation, has proposed a novel preventive treatment approach targeting individuals diagnosed early with hereditary prion diseases. This approach involves the use of ASOs directed against the *PRNP* gene (Vallabh et al., 2020). Symptomatic manifestation in mutation carriers usually occurs in late adulthood, presenting an opportunity for early therapeutic intervention to delay or prevent disease progression. Previous studies by Vallabh et al. have demonstrated in mice models that prophylactic administration of ASOs via intraventricular injection can reduce the deposition of pathogenic PrPSc and extend survival, even when treatment is initiated at the onset of clinical symptoms (Raymond et al., 2019). Additionally, they have proposed an alternative endpoint for predicting clinical benefits, such as measuring PrPC concentration in the brain. For some rare and refractory diseases, the United States Congress has agreed to adopt an accelerated approval strategy proposed by the FDA.^1^ This means that the FDA can expedite the approval of new therapies for these diseases to meet the urgent needs of patients.

The advantage of genetic prion diseases lies in the fact that a single gene encodes a single protein. Additionally, the unique gene target and verification though *in vivo* experiments make gene knockdown treatment of genetic prion diseases an ideal test case for leveraging predictive genetics to rewrite the future. These involves shifting therapeutic intervention upstream, beyond early symptoms or preclinical pathology, toward genetically informed primary prevention (Vallabh et al., 2020). The safety of PRNP gene knockout has been validated through early animal experiments. For instance, PRPN knockout mice display complete resistant to prion infection and exhibit normal development and behavior (Büeler et al., 1993). Lastly, Sonia Vallabh is a *PRNP* mutation carrier, and in the face of incurable diseases, she did not wait for fate but took a proactive stance in innovative clinical trials for genetic prion diseases. Vallabh's courage and gallantry are truly admirable (Aguzzi and Frontzek, 2020).

### 3.2 RNA interference, a completely suitable trial in presymptomatic mutation carriers

RNA interference (RNAi), emerging as a powerful gene treatment for neurological conditions, is a naturally occurring, highly conserved mechanism of gene silencing in eukaryotes (White and Mallucci, 2009). It is triggered by the presence of double-stranded RNA (dsRNA, exogenously introduced into cells such as viral RNA) or microRNA (miRNA, which endogenously regulate gene expression) (Zamore et al., 2000). Short interfering RNA (siRNA) is derived from long exogenous dsRNA which is recognized by Dicer and cut into short 21–23 nucleotide sequences. Both siRNA and miRNA interact with a multi-protein RNA-induced silencing complex (RISC); therefore, siRNA can mediate the mRNA degradation of targeted gene transcription, whereas miRNA directly lead to targeted gene silencing, both of which ultimately prevent the translation of pathogenic proteins (Figure 2) (Hutvagner and Zamore, 2002; Doench et al., 2003; Zeng et al., 2003). In 2006, Andrew Z. Fire and Craig C. Mello were awarded the Nobel Prize in Medicine for their contributions to the discovery of RNAi (The Nobel Prize in Physiology or Medicine, 2006), which opened numerous novel opportunities for developing various treatments for human diseases via RNAi (Kong, 2006).

Pfeifer et al. (2006) reported that lentivector-based anti-PrPC with short hairpin RNA (shRNA) stably knocked down PrPC and effectively suppressed prion replication in murine neuroblastoma cells; however, none of these studies used prion-infected animals. Later, White et al. (2008) reported the first case of using lentiviral mediated RNAi to effectively reduce the expression of PrPC in mice with prion disease, significantly prolonging the survival time. RNAi expressed by lentivectors also has been used to reduce PrPC levels in goats and cattle (Golding et al., 2006). Moreover, no obvious abnormality was found in these animal models that were treated with lentivector-based RNAi therapies, indicating that, at least in the animal model system, lentiviral mediated RNAi is well tolerated. These data provide an effective approach for neurodegenerative diseases and suggest that RNAi has therapeutic potential for individuals carrying pathogenic *PRNP* mutations to delay or inhibit the occurrence of diseases.

ASOs and RNAi indirectly decrease the pathogenic PrPSc by reducing PrPC levels. PrP-Fc2, a dimer with high solubility and stability, directly inhibits PrPSc replication and delays prion diseases (Meier et al., 2003). The Aguzzi team utilized lentiviral gene transfer to deliver PrP-Fc2 to the brains of prion-infected mice. Their research revealed a 41% extension in survival rates when the treatment was administered before prion inoculation or 14% when given 30 days after prion inoculation. These findings suggest that somatic gene transfer of prion antagonists may represent effective for prophylaxis and treatment of prion diseases (Genoud et al., 2008). While gene therapy using lentiviral vectors has demonstrated promising effects in laboratory and animal models, it is crucial to emphasize that before applying any gene therapy approach to humans, extensive clinical trials and research are necessary to ensure safety and effectiveness.

## 4 Advanced targeted protein degradation therapies

**Figure 3:**
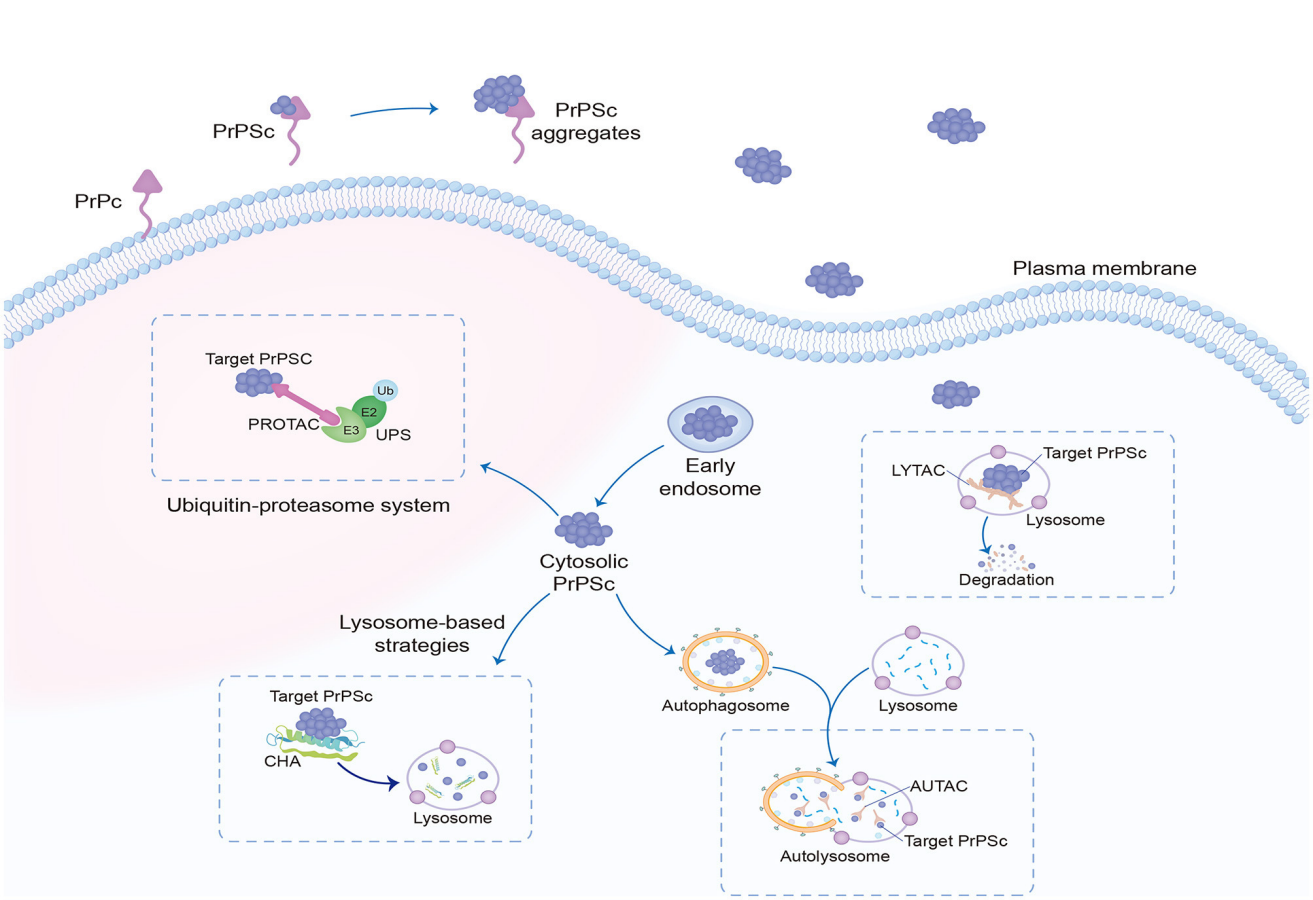
Targeted protein degradation. Targeted protein degradation is potential intracellular therapeutic points of prion clearance, including modulators of the UPS, as well as enhancers of autophagy and of lysosomal function. The most researched dependent UPS are PROTAC, and LYTAC, AUTAC, and CHA depend on lysosomal degradation. UPS, ubiquitin–proteasome system; PROTAC, proteolysis-targeting chimeras; LYTAC, lysosome-targeting chimera; AUTAC, autophagy-targeted chimera; CHA, chaperone-mediated autophagy.

In recent years, the development of targeted protein degradation (TPD) has generated tremendous excitement in the field of small-molecule drugs, including proteolysis-targeting chimeras (PROTAC) dependent on the ubiquitin–proteasome system (UPS) (Sakamoto, 2001) and lysosome-based TPD strategies, such as lysosome-targeting chimera (LYTAC) (Banik et al., 2020; Ahn et al., 2021; Zhou et al., 2021), autophagy-targeted chimera (AUTAC) (Takahashi et al., 2019; Ji et al., 2022), chaperone-mediated autophagy (CHA)-based degrader (Bourdenx et al., 2021), and other degradation methods (Figure 3). TPD modalities are capable of eliciting event-driven pharmacology to degrade previously undruggable proteins at relatively low concentrations, with a lower risk of off-target-based side effects (Lin et al., 2023).

CHA is a selective degrader neurodegeneration-related protein of the lysosomal pathway, and the most studied molecular chaperone is heat shock protein (HSP). HSPs are activated under endogenous and exogenous stressors, such as high temperature, toxicants, infection, or ischemia, to help cells maintain normal physiological activities. In parallel with the decrease in the effectiveness of the HSPs system with age, the ability of the body to clear pathological proteins decreases and the probability of degenerative disease increases with age. Mays et al. (2019) reported that prion disease was accelerated in mice lacking stress-induced HSP70, In contrast to PrPSc in mammals, PrPSc is proteinase sensitive in flies. Therefore, Fernandez-Funez et al. (2009) and Thackray et al. (2022) demonstrated that RNAi knockdown of HSP70 gene expression in flies model with prions enhanced neurotoxicity, whereas overexpression prevented the accumulation of PrPSc and protected against neurodegeneration toxicity. The mentioned TPD therapies primarily targets intracellular PrPSc, while another investigated target is the release of PrPC on the cell membrane. Shedding PrP (sPrP), a PrPC fragment, involves proteolytic cleavage and extracellular release mediated by the metalloproteinase ADAM10 (Taylor et al., 2009; Altmeppen et al., 2011). Research findings suggest that sPrP plays a neuroprotective role in protein misfolding diseases. The overexpression of ADAM10 in mice led to a reduction in PrPC levels within the brain. Furthermore, in mice with moderate ADAM10 overexpression, the incubation time following scrapie infection exhibited a significant increase (Endres et al., 2009; Altmeppen et al., 2015). Hence, employing a substrate-specific approach to induce ADAM10-mediated shedding of PrPC could represent a promising avenue for therapeutic interventions against prion diseases (Linsenmeier et al., 2021).

TPD therapies represent a promising strategy for addressing neurodegenerative disorders and have widely studied in a range of conditions, including AD, PD, Huntington's disease (HD) and frontotemporal dementia (FTD) (Fang et al., 2022). However, despite its potential therapeutic implications, TPD therapies encounter several significant challenges that must be addressed for successful clinical translation. These include issues such as limited solubility, suboptimal permeability across the blood-brain barrier, off-target effects leading to potential toxicity, and metabolic instability of the targeting ligands or degraders. Overcoming these obstacles requires rigorous optimization of TPD agents, including the design of highly specific degraders, enhancement of pharmacokinetic properties, and thorough evaluation of safety profiles in preclinical models. Furthermore, innovative delivery strategies and novel chemical modifications may be necessary to improve the overall efficacy and selectivity of TPD approaches in the context of neurodegenerative disorders. For instance, the evolution of monomeric degraders, which possess smaller molecular weights, enhances their capability to traverse the blood-brain barrier more efficiently (Li et al., 2021). Currently, investigations are underway to explore the integration of nanotechnology-based smart delivery systems. These systems aim to synergistically enhance the solubility, permeability, and targeting capability of TPD drugs, thereby reducing the risk of off-target toxicity (Zhong et al., 2024).

## 5 Using stem cell technology as a tool to repair damaged neurons

**Figure 4:**
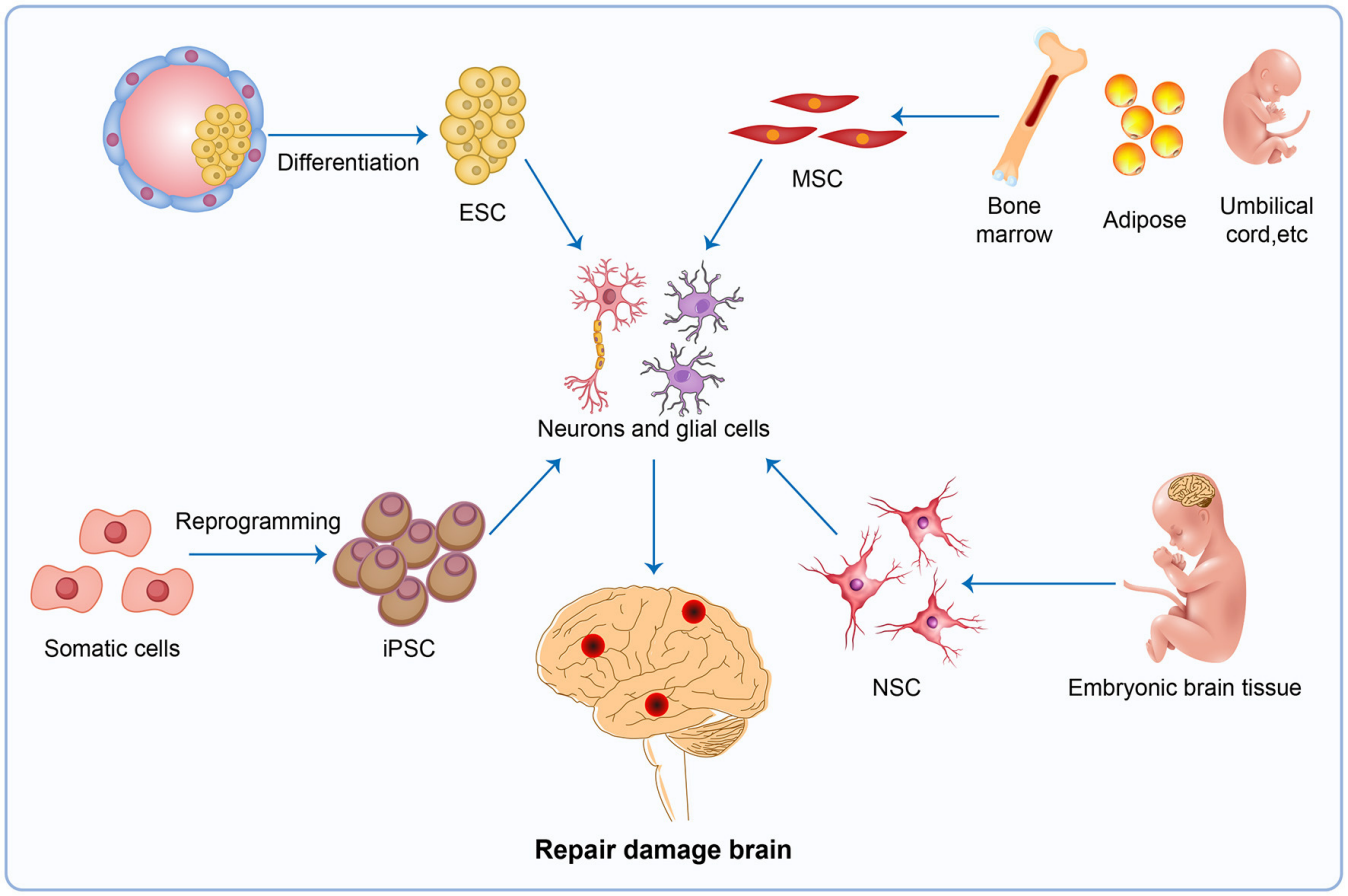
Stem cell therapy. The injured brain tissue maybe be repaired by stem cell therapy, a potential therapy in neurodegenerative disease. The most promising avenues for stem cell therapy include ESC, MSC, iPSC and NSC. ESC, embryonic stem cells; MSC, mesenchymal stem cells; iPSC, induced pluripotent stem cells; NSC, neuron stem cells.

Almost all current therapeutic strategies have focused on preventing or clearing infectious prion particles; however, some researchers have addressed stem cell therapies to repair damaged neurons caused by misfolded pathological prions. The most frequently researched stem cell therapy includes embryonic stem cells (ESCs), mesenchymal stem cells (MSCs), induced pluripotent stem cells (iPSCs), and neuron stem cells (NSCs) (Figure 4). Stem cell-based therapy has shown great prospects in neurodegenerative diseases including PD (Kordower et al., 1995; Freed et al., 2001), HD (Curtis et al., 2003), and amyotrophic lateral sclerosis (ALS) (Clowry et al., 1991; Garbuzova-Davis et al., 2002; Kerr et al., 2003), leading to the initiation of clinical trials at various stages of progress. Additionally, Relaño-Ginés et al. (2011) demonstrated its effectiveness in prion disease animal models by revealing that intracerebral transplantation of fetal neural stem cells significantly prolonged both incubation and survival times. Recent advances offer hope for the development of stem cell therapies in human neurodegenerative disorders, as neurons suitable for transplantation can be generated from stem cells in culture, and the adult brain produces new neurons from its own stem cells in response to injury (Lindvall et al., 2004).

Progress in stem cell technology shows that tissue regeneration is very promising; however, it is worth noting that it would be premature to launch clinical trials to use stem cells for treating neurological disorders. The control of stem cell proliferation and differentiation into specific phenotypes and the prevention of tumor formation bear the brunt of things. Furthermore, it may be difficult to find the best source of stem cells for nerve regeneration; ethical, immune rejection (Takahashi and Yamanaka, 2006), and the risk of causing tumor (teratoma) (Sonntag et al., 2018) should also be considered. It's important to keep in mind that while the neurobiological mechanisms behind stem cell therapy are exciting, the practical use of stem cells in clinical settings will depend on their ability to provide patients with neurological disorders substantial and lasting improvements in quality of life, all while ensuring safety (Lindvall and Kokaia, 2006).

## 6 Conclusions and future perspectives

Prion disease is recognized as one of the rapidly progressing neurodegenerative diseases. Despite significant efforts in this area, there is currently no fully effective treatment available. It is worth recalling that cancer chemotherapy was also minimally effective 40 or 50 years ago. However, through persistent determination and repeated efforts, malignancies can now be expected to be cured solely through drug treatments.

In this study, we have examined numerous emerging technologies in the context of prion diseases, including immunotherapy, gene therapy, targeted protein degradation therapies, and stem cell technology. Additionally, we have outlined the functional mechanisms of current prion diseases therapeutic pathways in four figures.

The emergence of these novel therapeutic targets and research findings, whether *in vivo* or *in vitro*, is indeed promising, yet they are still in their early stages. When transitioning drugs from preclinical research to clinical research, considerations such as toxicity, efficacy, and pharmacokinetics are paramount. Moreover, conducting double-blind, randomized, placebo-controlled, multicenter trials on large patient cohorts poses significant challenges due to the low prevalence of human prion diseases, the absence of valid alternative endpoints, the rapid progression of the disease, and its heterogeneity. Despite formidable challenges, there is a growing scientific interest in novel therapeutic approaches for this disease. These findings may suggest a shift in the medical paradigm, transitioning from relentless efforts focused on externally introducing drugs to selectively recruiting autoimmune cells to combat brain diseases, where the aggregation of abnormal proteins may only represent a fraction of the overall picture.

## Author contributions

FL: Writing—original draft, Writing—review & editing. WL: Conceptualization, Data curation, Formal analysis, Investigation, Software, Validation, Writing—review & editing. LL: Funding acquisition, Methodology, Project administration, Resources, Supervision, Visualization, Writing—review & editing.

## Footnotes

^1^https://www.fda.gov/patients/fast-track-breakthrough-therapy-accelerated-approval-priority-review/accelerated-approval (accessed February 3, 2024).
